# Non-Apoptotic Caspase Activity Preferentially Targets a Novel Consensus Sequence Associated With Cytoskeletal Proteins in the Developing Auditory Brainstem

**DOI:** 10.3389/fcell.2022.844844

**Published:** 2022-03-07

**Authors:** Forrest Weghorst, Yeva Mirzakhanyan, Kiersten L. Hernandez, Paul D. Gershon, Karina S. Cramer

**Affiliations:** ^1^ Department of Neurobiology and Behavior, UC Irvine, Irvine, CA, United States; ^2^ Department of Molecular Biology and Biochemistry, UC Irvine, Irvine, CA, United States

**Keywords:** auditory brainstem, neural development, caspase, non-apoptotic, proteomics, cytoskeleton

## Abstract

The auditory brainstem relies on precise circuitry to facilitate sound source localization. In the chick, the development of this specialized circuitry requires non-apoptotic activity of caspase-3, for which we previously identified several hundred proteolytic substrates. Here we tested whether the sequence of the caspase cleavage site differentially encodes proteolytic preference in apoptotic and non-apoptotic contexts. We constructed a consensus sequence for caspase activity in the non-apoptotic chick auditory brainstem comprising the four residues N-terminal to the cleavage site: IX(G/R)D↓ where X represents no significant enrichment and ↓ represents the cleavage site. We identified GO terms significantly enriched among caspase substrates containing motifs found in the above consensus sequence. (G/R)D↓ was associated with the term “Structural Constituent of Cytoskeleton” (SCoC), suggesting that SCoC proteins may be specifically targeted by caspase activity during non-apoptotic developmental processes. To ascertain whether this consensus sequence was specific to the non-apoptotic auditory brainstem at embryonic day (E) 10, we used protein mass spectrometry of brainstems harvested at a time when auditory brainstem neurons undergo apoptotic cell death (E13). The apoptotic motif VD was significantly enriched among E13 cleavage sites, indicating that motif preference at the P2 subsite had shifted toward the canonical caspase consensus sequence. Additionally, Monte Carlo simulations revealed that only the GD motif was associated with SCoC substrates in the apoptotic auditory brainstem, indicating that GD encodes specificity for SCoC proteins in both non-apoptotic and apoptotic contexts, despite not being preferred in the latter. Finally, to identify candidate human non-apoptotic consensus sequences, we used Monte Carlo analyses to determine motifs and motif pairs associated with SCoC caspase substrates in the Degrabase, a database of cleavage sites in human apoptotic cell lines. We found 11 motifs significantly associated with SCoC proteolysis, including IXXD and GD. We employed a stepwise method to select motif pairs that optimized SCoC specificity for a given coverage of SCoC cleavage events, yielding 11 motif pairs likely to be preferred in SCoC-directed human non-apoptotic caspase consensus sequences. GD + IXXD was among these motif pairs, suggesting a conservation of non-apoptotic consensus sites among vertebrates.

## Introduction

The auditory system has two main tasks: identifying sounds and determining sound source location. The first task involves encoding features of the sound itself, such as frequency, intensity, and timbre (Agus et al., 2019). However, the second task is more complex because sounds do not come with explicit information regarding their source’s location. Instead, the auditory brainstem models the sound landscape by integrating differences in sound intensity and arrival time between the ears, known as interaural level differences (ILDs) and interaural time differences (ITDs), respectively (Carr and Konishi, 1990; Overholt et al., 1992; Schroger, 1996; Hyson, 2005). Highly specialized circuitry is necessary to detect microsecond-level ITDs, which are spatially represented in the auditory brainstem according to both ITD magnitude and sound frequency (Köppl and Carr, 2008; Ohmori, 2014). This dual specificity presents a unique neurodevelopmental challenge to the processes that regulate axon guidance and synapse formation in the ITD pathway. It is unsurprising, therefore, that neurodevelopmental disorders that alter circuit development are commonly accompanied by anatomical and functional deficits in the auditory brainstem (Talge et al., 2018; Smith et al., 2019; McCullagh et al., 2020; Chen et al., 2021; Miron et al., 2021; Talge et al., 2021). The auditory brainstem is thus an ideal model system for studying the development of ultra-precise neural circuitry.

We have addressed the molecular mechanisms behind formation of the embryonic chick auditory brainstem, specifically in the role of non-apoptotic caspase activity in the assembly of the chick ITD circuit. While caspases are classically associated with programmed cell death (Hengartner, 2000; Elmore, 2007; Fuchs and Steller, 2011), their non-lethal roles in cell differentiation pathways and morphological change are now receiving overdue recognition (Schwerk and Schulze-Osthoff, 2003; Galluzzi et al., 2012; Unsain and Barker, 2015; Nakajima and Kuranaga, 2017). The nervous system is particularly well-equipped to use non-apoptotic caspase activity with little risk of accidental death (Campbell and Holt, 2003; Campbell and Okamoto, 2013; Wang and Luo, 2014; Gu et al., 2017; Hollville and Deshmukh, 2017; Mukherjee and Williams, 2017; Kellermeyer et al., 2018; Espinosa-Oliva et al., 2019; Nguyen et al., 2021). Neurons are remarkably resistant to injury, and the primary sites of morphological change in neurons (axons and dendrites) are often far removed from critical targets of apoptosis in and around the nucleus. We previously reported (Rotschafer et al., 2016) that expression of cleaved caspase-3 ascends the ITD pathway, appearing first in axons of the auditory nerve (AN) on embryonic days (E) 6-7; followed by expression in axons of the AN’s target, nucleus magnocellularis (NM) on E8-10; and finally in dendrites of NM’s target, nucleus laminaris (NL) on E10-12. Inhibition of caspase-3 activity with z-DEVD-fmk in cultures mimicking E6-9 elicited major defects in NM axon targeting and NL nucleus morphology on E10, prior to the appearance of TUNEL labeling in NM and NL cell bodies (Rotschafer et al., 2016), and to the onset of developmental cell loss in the auditory brainstem (Rubel et al., 1976). Caspase-3 thus mediates development of the auditory brainstem in the absence of cell death, suggesting a non-apoptotic function of caspase activity in circuit assembly.

To understand these developmental roles, it is important to consider how non-apoptotic caspase-3 activity facilitates neurodevelopment without causing cell death. In a recent study (Weghorst et al., 2020), we identified several hundred non-apoptotic caspase-3 substrates, which differed from apoptotic caspase substrates in both protein content and gene ontology (GO) term annotation. This distinction suggested that caspase-3 activity is actively redirected toward a novel, non-lethal substrate repertoire in the chick auditory brainstem, but the mechanism for this change remained unclear. Multiple substrate features enable caspase recognition, including the amino acid sequence surrounding the potential cleavage site, as well as substrate-caspase interactions at allosteric protein regions, or exosites, that favorably place cleavage sites near the caspase active site (Talanian et al., 1997; Fischer et al., 2003; Timmer and Salvesen, 2007; Julien and Wells, 2017). The former category was seemingly refuted by the discovery that many *in vivo* caspase cleavage sites are cleaved with high efficiency despite their substantial deviation from the preferred cleavage site sequence, as identified via positional scanning substrate combinatorial libraries of synthetic peptides (Thornberry et al., 1997; Stennicke et al., 2000; Fischer et al., 2003; Timmer and Salvesen, 2007; Crawford et al., 2013; Julien and Wells, 2017). Exosite interactions provided a more parsimonious explanation for the cleavage of suboptimal sites, with a subsidiary role for cleavage site sequence in specific substrate recognition, binding, and proteolysis (Dagbay et al., 2014). However, a dominant role for exosites in facilitating diverse cleavages within the context of apoptosis does not preclude a role for cleavage site sequences in enabling a switch in substrate preference for non-apoptotic functions.

In the present study, we hypothesized that the preferred amino acid sequence N-terminal of the caspase cleavage site is shifted from the apoptotic consensus sequence (typically DEVD↓, where ↓ represents the cleavage site) to an alternative consensus sequence that is found in non-apoptotic substrates in the auditory brainstem. We showed that the non-apoptotic auditory brainstem caspase degradome involves a non-canonical consensus sequence, IX(G/R)D. Proteins cleaved at this consensus sequence were enriched for the GO term “Structural Constituent of Cytoskeleton” (SCoC), suggesting that the non-apoptotic consensus sequence allows caspases to preferentially target cytoskeletal proteins. We characterized the proteome of the apoptotic chick auditory brainstem and discovered the apoptotic consensus sequence (K/M)VD, suggesting that the non-apoptotic consensus sequence truly represents a switch in preference. Finally, we used the Degrabase (Crawford et al., 2013) to predict likely candidates for an SCoC-directed non-apoptotic consensus sequence in humans.

## Methods

### Data Sources

Non-apoptotic chick auditory brainstem peptidomic data were sourced from our previous publication (Weghorst et al., 2020). Briefly, eggs from a hybrid flock of White Leghorn males and Rhode Island Red females (AA Lab Eggs) were incubated in a rotating shelf incubator at 37.5°C for 72 h. Contents were removed from the shell, transferred to *ex ovo* cultures on embryonic day (E) 3, and the cultures were incubated until 7 days *in vitro* (DIV), corresponding to approximately E10. Brainstems were dissected, snap-frozen and stored at −80°C in 3 biological replicates of 2 brainstems each. Nano-LC-MS/MS was used to characterize the peptidome of the samples. Only data from control samples (not caspase-3-inhibited samples) were used for the present study. Human apoptotic substrate data were sourced from Degrabase (Crawford et al., 2013). Chick apoptotic peptidomic data were generated from E13 auditory brainstem tissue. Eggs from a hybrid flock of White Leghorn males and Rhode Island Red females (AA Lab Eggs) were incubated in a rotating shelf incubator at 37.5°C for 13 days. The auditory portion of the brainstem was dissected, snap-frozen and stored at −80°C in three biological replicates, each containing two pooled brainstems.

The three E13 samples were each disaggregated in 70% formic acid for 72 h at room temperature with constant shaking and occasional ultrasonication in a cuphorn sonicator. One crystal of cyanogen bromide (CNBr) was added to each sample, followed by overnight incubation in the dark. Samples were evaporated to dryness in a Speedvac vacuum concentrator, then redissolved in 8 M urea, 100 mM triethyl ammonium bicarbonate (TEAB; pH 8.0), 10 mM tris(2-carboxyethyl)phosphine, diluted to 6 M urea using 100 mM TEAB, and digested overnight at 37°C with LysC (Promega - R-LysC; enzyme:substrate mass ratio = 1:100). Samples were diluted to 1 M urea with 0.1 M TEAB, then trypsinized (Promega - Trypsin Gold; enzyme:substrate mass ratio = 1:100). The resulting peptides were isolated using a stacked C18/SCX STAGE tip (Rappsilber et al., 2007) eluting with 54 mM, 73 mM, 100 mM, 130 mM, 176 mM, and 395 mM ammonium acetate in 20% acetonitrile, 0.5% formic acid (FA), with a final elution in 5% ammonium hydroxide, and 80% acetonitrile. Dried elutions were redissolved in 0.1% FA in water. The resulting samples were injected sequentially to an LTQ Orbitrap Velos Pro via an Easy-nLC 1,200, developing a gradient from 5–23% B (93% CH_3_CN in water) over 115 min then to 35% B over 20 min, 0.25 μl/min. In each precursor scan (FTMS; 60,000 resolution) up to the 15 most intense ions with intensity value of >2000, excluded +1 charge state and dynamic exclusion (repeat count = 1, repeat duration = 30 s, exclusion list size = 500, exclusion duration = 40 s) were selected for MS2 (FTMS; 7,500 resolution) via HCD fragmentation. Data from groups of seven elutions were combined and searched using Mascot 2.7 against the *Gallus* (taxon ID 9031) UniProt reference proteome plus a database of common contaminants, with allowable charge states of +2 to +4 and allowing CNBr-trypsin cleavage specificity, as an error-tolerant search (which allows semiCNBr-trypsin specificity), and with precursor and product mass tolerances of ±20 ppm and ±20 mmu respectively. Data were thresholded at significance (*p*) < 0.05. The mass spectrometry proteomics data have been deposited to the ProteomeXchange Consortium via the PRIDE partner repository with the dataset identifier PXD030697 (Perez-Riverol et al., 2019).

### Consensus Sequence Analysis

We previously used IceLogo (Maddelein et al., 2015) to generate a cleavage site consensus sequence for non-apoptotic caspase-3 substrates in the chick auditory brainstem (Weghorst et al., 2020). This consensus sequence (DHRD↓) was created using peptides both N- and C-terminal to the cleavage site, but some shortcomings with this method led us to consider an alternative approach for the present study. First, IceLogo assumes a normal distribution for amino acid frequencies to avoid assumptions about any subsite in the input sequences. A methodology more tailored to caspases would account for the fact that they primarily cleave C-terminal to aspartate residues (Seaman et al., 2016). This preference suggests a hypergeometric distribution. Second, the inclusion of peptides N-terminal to the cleavage site while creating an N-terminal consensus sequence is problematic because motif enrichment due to preferential proteolysis cannot be distinguished from enrichment due to greater MS detectability of peptides with specific amino acids. We therefore aimed to explore the consensus sequence for non-apoptotic caspase activity in the auditory brainstem using a more appropriate statistical method to analyze only peptides C-terminal to the caspase cleavage site.

The cleavage site consensus sequence for non-apoptotic auditory brainstem caspase activity was derived by subsite enrichment analysis of the 20 amino acids at the P4-P2 positions of caspase cleavage sites (Schechter and Berger, 1967). Caspase cleavage sites were identified by detection of peptides with a D↓X terminus (where ↓ represents a cleavage site and X represents the first amino acid of the C-terminal peptide) that appeared in at least one control sample of our prior study (Weghorst et al., 2020). Cleavage sites from proteins in the same family with identical P4-P4’ sequences were treated as a single cleavage event. For each P4-P2 motif, a two-tailed hypergeometric test (Table 1) was used to compare the frequency of the motif in observed caspase cleavage sites (k_Total_/n_Total_) with the frequency of the motif relative to all aspartate residues in substrate sequences (K_Total_/N_Total_). The mid-*p*-value variant of the hypergeometric test was used to compute *p*-values (Lancaster, 1961), and a two-stage linear step-up procedure (Benjamini et al., 2006) was used to control the false discovery rate (FDR) of the 60 hypergeometric tests. This method was also used to generate a consensus sequence for Degrabase.

**TABLE 1:** Definition and derivation of variables used in hypergeometric tests for each P4-P2 motif in caspase consensus sequences.

Variable	Definition	Derivation
k	Count of sites cleaved at the motif	$k_{\mathrm{Total}}={\sum_{\mathrm{i=1}}^{R}\left(\sum_{\mathrm{j=1}}^{P}k_{j}\right)}_{i}$
n	Count of sites cleaved	$n_{\mathrm{Total}}={\sum_{\mathrm{i=1}}^{R}\left(\sum_{\mathrm{j=1}}^{P}n_{j}\right)}_{i}$
K	Count of the motif in substrate sequence	$K_{\mathrm{Total}}={\sum_{\mathrm{i=1}}^{R}\left(\sum_{\mathrm{j=1}}^{P}k_{j}\right)}_{i}$
N	Count of aspartates in substrate sequence	$N_{\mathrm{Total}}={\sum_{\mathrm{i=1}}^{R}\left(\sum_{\mathrm{j=1}}^{P}N_{j}\right)}_{i}$

R, Count of replicates. P, Count of cleaved proteins.

### Functional Annotation Analysis

The Database for Annotation, Visualization, and Integrated Discovery (DAVID) Bioinformatics Resource 6.8 (Huang et al., 2009a; Huang et al., 2009b) was used to compare the frequency of GO terms among caspase substrates with P2 subsites that resemble the cleavage site consensus sequence to the frequency of GO terms among all caspase substrates in the auditory brainstem. Hypergeometric mid-*p*-values were calculated for each GO term (Lancaster, 1961), and a two-stage linear step-up procedure (Benjamini et al., 2006) was used to control the false discovery rate (FDR) for each of the three categories of GO terms (Cellular Component, Biological Process, and Molecular Function).

### “Structural Constituent of Cytoskeleton” Subsite Analysis

R (v. 3.6.1) was used to conduct Monte Carlo simulations to compare the observed number of SCoC cleavage events attributable to each P2-P4 motif with the expected distribution of SCoC cleavage events attributable to that motif, based on random proteolysis at the overall frequency of each motif in each biological replicate. Within each replicate, the probability that each SCoC substrate was cleaved at each motif was calculated as follows:

$$Pr\left(Substrate\;cleaved\;at\;motif\right)=1-\overset{S-1}{\underset{i=0}{\Pi }}{\left(1-\frac{\Sigma_{\mathrm{j=}1}^{P}\;k_{j}}{\left(\Sigma_{\mathrm{j=}1}^{P}\;n_{j}\right)\mathrm{-i}}\right)}_{i}$$

In this equation, P denotes the count of proteins cleaved in the replicate; S denotes the count of sites cleaved in the SCoC protein in the replicate; and definitions for k and n are shown in Table 1.

Protein-cleavages were simulated at the observed frequency of each motif by comparing each SCoC protein’s Pr(*Substrate cleaved at motif*) to a randomly generated variable *x,* where x ∈ R and x ∈ [0,1).

$$If\;x<\;Pr\left(Substrate\;cleaved\;at\;motif\right):C_{j}=1$$

$$If\;x\geq \;Pr\left(Substrate\;cleaved\;at\;motif\right):C_{j}=0$$

Expected count of total protein-cleavages at each motif was then calculated in the same way as the observed count of protein cleavages:

$$C_{\mathrm{Total}}={\sum_{\mathrm{i=1}}^{R}\left(\sum_{\mathrm{j=1}}^{\mathrm{SCoC}}C_{j}\right)}_{i}$$

Here, C denotes the count of protein-cleavages; SCoC denotes the count of cleaved SCoC proteins; and R denotes the count of replicates.

A *p*-value for each motif was computed by the mid-*p*-value method for discrete distributions (Lancaster, 1961). Fold enrichment for each motif was computed as observed C_Total_ divided by the mean of the expected distribution of C_Total_. The same method was used to assess combinations of motifs in the human apoptotic proteome.

## Results

### Non-Apoptotic Caspase Cleavage Sites in the Chick Auditory Brainstem Are Enriched for the Motifs IXXD, RD, and GD

**FIGURE 1:**
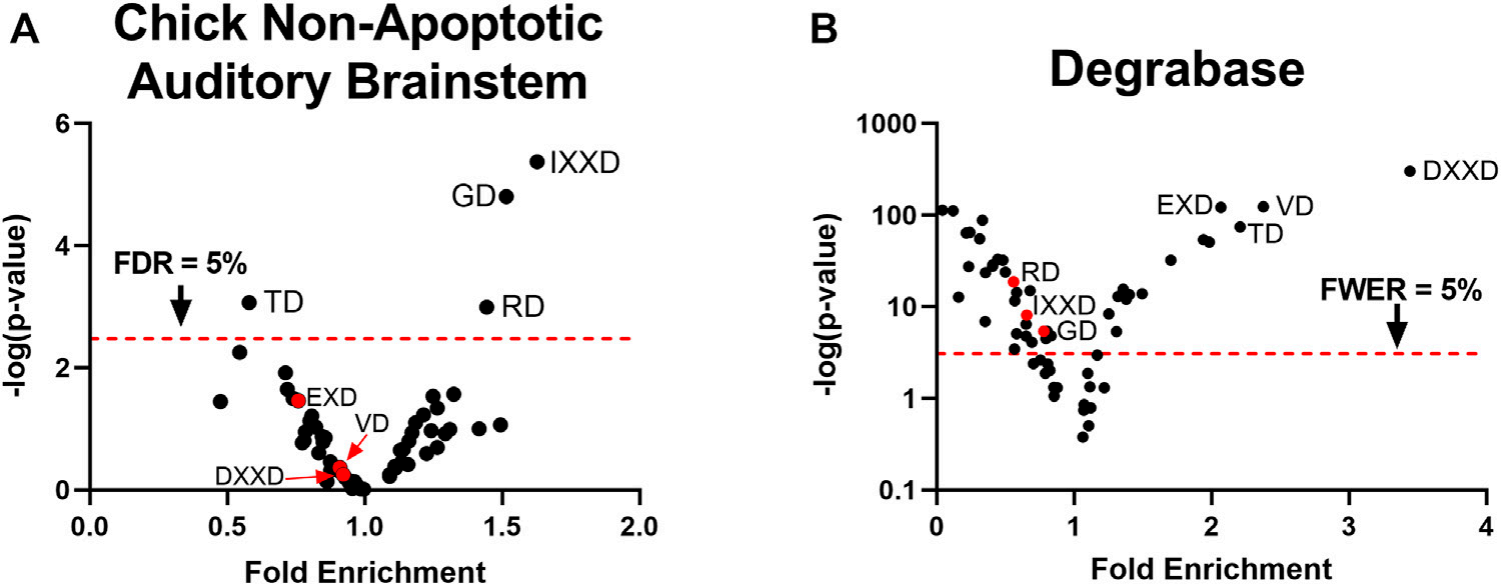
Caspase activity prefers a non-canonical consensus sequence in the non-apoptotic chick auditory brainstem. **(A)** Hypergeometric tests were used to compare observed and expected frequencies of each of the 60 P4-P2 motifs at aspartic cleavage sites in the chick auditory brainstem. Expected frequencies were estimated as the proportion of each motif preceding all aspartates in the protein sequences of substrates, adjusted for the number of replicates in which each substrate was cleaved. We found a positive enrichment for the motifs IXXD, GD, and RD, yielding a consensus sequence of IX(G/R)D. **(B)** As a point of reference for the non-apoptotic consensus sequence, we used the same method to compare observed and expected frequencies of P4-P2 motifs at aspartic cleavage sites in the Degrabase, a published database of neo-N-termini from apoptotic human cell lines. As expected, the Degrabase exhibited a consensus sequence of DEVD, the preferred P4-P1 sequence of executioner caspase-3 and -7. By contrast, the Degrabase showed a negative enrichment for the consensus sequence of the non-apoptotic chick auditory brainstem, suggesting that caspase activity in the chick auditory brainstem is directed toward sites with a P4-P2 sequence distinct from that observed during apoptosis. FDR: False Discovery Rate, derived by Benjamini, Krieger and Yekutieli correction. FWER: Family-Wise Error Rate, derived by Bonferroni correction.

Using label-free peptidomic data from our prior study (Weghorst et al., 2020), we constructed a cleavage site consensus sequence for non-apoptotic caspase activity in the embryonic chick auditory brainstem. We filtered for peptides with an N-terminal D↓X terminus, where D represents the P1 aspartate, ↓ represents a cleavage site, and X represents the N-terminus of the peptide. This criterion yielded peptides corresponding to 655 distinct cleavage sites from 365 proteins (Supplementary Data S1). We used hypergeometric tests to compare the frequency of each single-residue motif in the P2-P4 subsites of these cleavage sites (Schechter and Berger, 1967) to the motif’s frequency relative to all aspartate residues in the sequences of cleaved proteins. After correction for multiple comparisons, we found that three motifs were enriched above chance: IXXD (Fold enrichment: 1.63; *p* = 4.3 × 10^–6^; *q* = 2.5 × 10^–4^), GD (Fold enrichment: 1.52; *p* = 1.6 × 10^–5^; *q* = 4.6 × 10^–4^), and RD (Fold enrichment: 1.44; *p* = 1.0 × 10^–3^; *q* = 0.015), corresponding to the consensus sequence IX(G/R)D (Figure 1A). We applied the same method to a human apoptotic peptidome, Degrabase (Crawford et al., 2013), both as a proof of concept for this technique of identifying caspase consensus sequences and as a point of comparison for the chick non-apoptotic consensus sequence. The Degrabase consensus sequence showed the expected enrichment for the executioner caspase cleavage site preference (DEVD↓), while also exhibiting significant de-enrichment for the motifs enriched in the non-apoptotic auditory brainstem (Figure 1B). Conversely, the apoptotic consensus sequence was non-significantly de-enriched in the non-apoptotic auditory brainstem (Figure 1A).

### Non-Apoptotic Caspase Substrates With Consensus-like Cleavage Sites Are Enriched for the GO Term “Structural Constituent of Cytoskeleton”

**FIGURE 2:**
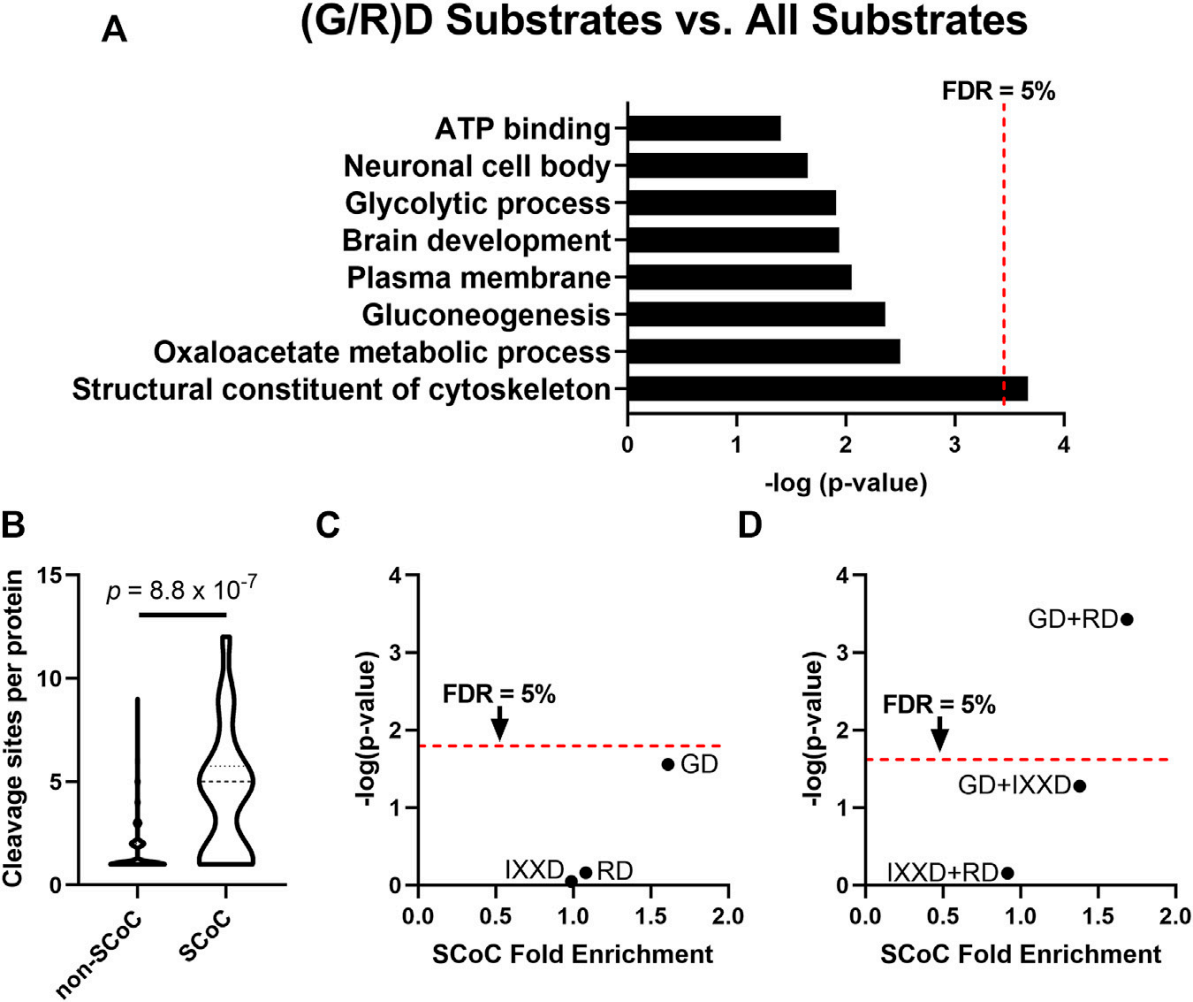
The P2 position of the non-apoptotic auditory brainstem caspase consensus sequence is associated with substrates enriched for the GO term “Structural Constituent of Cytoskeleton” (SCoC). **(A)** The DAVID Bioinformatics Resource 6.8 was used to examine GO terms of non-apoptotic chick auditory brainstem substrates with at least one GD or RD site. These substrates with consensus-like sites were significantly more likely to have the GO term “Structural Constituent of Cytoskeleton” (SCoC) than were auditory brainstem caspase substrates as a whole. **(B)** “Structural Constituent of Cytoskeleton” substrates had a greater number of caspase cleavage sites per protein per replicate than other substrates (Mann Whitney U-test). This result suggests that an alternative method is needed to ensure that the enrichment of SCoC proteins among caspase substrates with consensus-like sites is not due to SCoC proteins having a large number of cleavage sites, any of which could be consensus-like. **(C)** For each motif in the non-apoptotic auditory brainstem consensus sequence, Monte Carlo analysis was used to simulate the number of SCoC proteins expected to be cleaved at least once at that motif in each replicate (denoted a “protein-cleavage”) based on each replicate’s overall frequency of each motif. Total protein-cleavage counts were obtained by summing the counts for each replicate. The distribution of expected protein-cleavage totals was compared to that of observed protein-cleavage counts to obtain the SCoC Fold Enrichment and *p*-value. Only the GD motif was enriched among SCoC substrates (*q* = 0.086), while RD and IXXD were cleaved at chance. **(D)** The three double-motif combinations of the non-apoptotic auditory brainstem consensus sequence were subjected to Monte Carlo analyses as in part C. The GD + RD motif combination was enriched among SCoC substrates even more than either of its constituent motifs (*q* = 7.8 × 10^–4^). FDR: False Discovery Rate, derived by Benjamini, Krieger, and Yekutieli correction.

We previously showed that caspase-3 is the most abundant caspase in the non-apoptotic auditory brainstem and is therefore likely responsible for most of the caspase cleavage sites we detected (Weghorst et al., 2020). This finding parallels the role of caspase-3 as the primary executioner caspase during apoptosis (Slee et al., 2001). However, caspase-3 has a conserved preference for DEVD (Thornberry et al., 1997; Stennicke et al., 2000; Grinshpon et al., 2019), so the discovery of the IX(G/R)D consensus sequence is surprising. One possible explanation for this novel caspase-3 preference is a difference in the S4 and S2 active site pockets (Fuentes-Prior and Salvesen, 2004), which receive the P4 and P2 substrate residues, respectively. We performed an additional analysis to identify characteristics of substrates with cleavage sites containing these novel consensus motifs at P2 and P4. We used DAVID Bioinformatics Resource 6.8 (Huang et al., 2009a; Huang et al., 2009b) to identify GO terms enriched among auditory brainstem caspase substrates containing cleavage sites with enriched motifs, compared to the background of all 365 caspase substrates. Because the different active site pockets play distinct roles in substrate selection, any difference in a caspase active site pocket will affect the preference for all substrate motifs in that pocket but not necessarily of the other pockets. Consequently, changes to the S2 pocket would likely mediate preference for both RD and GD, while separate changes to the S4 pocket would shift its preference to IXXD. In this functional annotation analysis, we therefore pooled the P2 motifs (RD and GD), which we investigated separately from the enriched P4 motif (IXXD). The 57 substrates with IXXD sites showed no significant GO term enrichment after correction for multiple comparisons (Supplementary Data S2). By contrast, the 101 substrates with RD or GD sites were enriched for a single GO term, “Structural constituent of cytoskeleton” (“SCoC”; Figure 2A). According to DAVID, all 6 SCoC substrates in our dataset had at least one RD or GD cleavage site (Fold enrichment: 3.55; *p* = 2.2 × 10^–4^; *q* = 0.032), suggesting that non-apoptotic caspase activity in the auditory brainstem prefers cleavage site motifs that are found in cytoskeletal proteins.

### SCoC Proteins Are More Likely to be Cleaved at the GD Motif and the Combined (G/R)D Motifs Than Expected by Chance

Two limitations in using DAVID to identify functional commonalities of consensus-like sites led us to refine our analysis. First, the chicken proteome is not as well-annotated as the proteomes of humans and more common model organisms, so chicken proteins often lack relevant GO terms. To correct this limitation, we manually searched the list of auditory brainstem caspase substrates for proteins with a human homolog that had the SCoC GO term. We identified three additional SCoC substrates, two of which had a GD cleavage site, bringing the fold enrichment of SCoC proteins among proteins with (G/R)D sites to 3.15 (*p* = 1.1 × 10^–4^, *q* = 0.016). Second, functional annotation analysis of proteins with consensus-like cleavage sites is biased toward substrates with greater numbers of cleavage sites. A single (G/R)D site qualifies a protein as having a consensus-like cleavage event, regardless of how many non-consensus-like sites the protein also has. Auditory brainstem SCoC proteins had substantially more cleavage sites per protein than non-SCoC proteins (Figure 2B; Mann-Whitney U-test, *p* = 8.8 × 10^–7^), so SCoC proteins are inherently more likely to be cleaved at any site.

We therefore used Monte Carlo analyses to determine whether SCoC proteins are cleaved at consensus-like sites at a frequency greater than chance. In each biological replicate, each distinct SCoC protein was randomly “cleaved” at each of its observed sites, assuming the probability of cleavage at a specific motif was equal to the observed frequency of that motif among all cleavage sites in the same replicate. The total number of SCoC protein-cleavages attributable to each motif was obtained by summing the protein-cleavages for all SCoC proteins in all replicates. The simulated distribution of SCoC protein-cleavages for each motif was then compared to the observed number of SCoC protein-cleavages for that motif to obtain a fold enrichment and *p*-value for the observation. We conducted this analysis for the 3 consensus motifs, as well as for the 3 double-motif combinations, and found that while SCoC proteins are cleaved at IXXD and RD sites at chance (Fold enrichment: 0.99 and 1.08; *p* = 0.90 and 0.69, respectively; *q* = 0.94 for each), proteolysis of SCoC proteins occurs at GD sites at a frequency greater than chance (Figure 2C; Fold enrichment: 1.61; *p* = 0.028; *q* = 0.087). For the double-motif analyses, GD + RD was enriched among SCoC proteins even more than GD or RD alone (Figure 2D; Fold enrichment: 1.69; *p* = 3.7 × 10^–4^, *q* = 7.8 × 10^–4^), suggesting complementarity of the two motifs in cleaving SCoC proteins. Indeed, 17 of the 20 observed SCoC protein-cleavages had at least one GD or RD site, a greater proportion than any other pair of motifs. GD + IXXD was enriched among SCoC cleavage sites to a lesser extent (fold enrichment: 1.38; *p* = 0.053; *q* = 0.055), while IXXD + RD cleavage events of SCoC proteins were not enriched (Fold enrichment: 0.92; *p* = 0.70; *q* = 0.49). These data indicate that the P2 subsite preference of non-apoptotic caspase activity in the chick auditory brainstem is directed toward a specific motif (GD) and a motif combination (GD + RD) that are overrepresented among cytoskeletal protein-cleavages. These data suggest that the preferred cleavage site sequence of non-apoptotic caspase activity causes preferential proteolysis of cytoskeletal substrates during auditory brainstem development.

### SCoC Cleavage Events in the Apoptotic Chick Auditory Brainstem Are Disproportionately Associated With the GD Motif, Despite Its Absence From the Apoptotic Consensus Sequence

**FIGURE 3:**
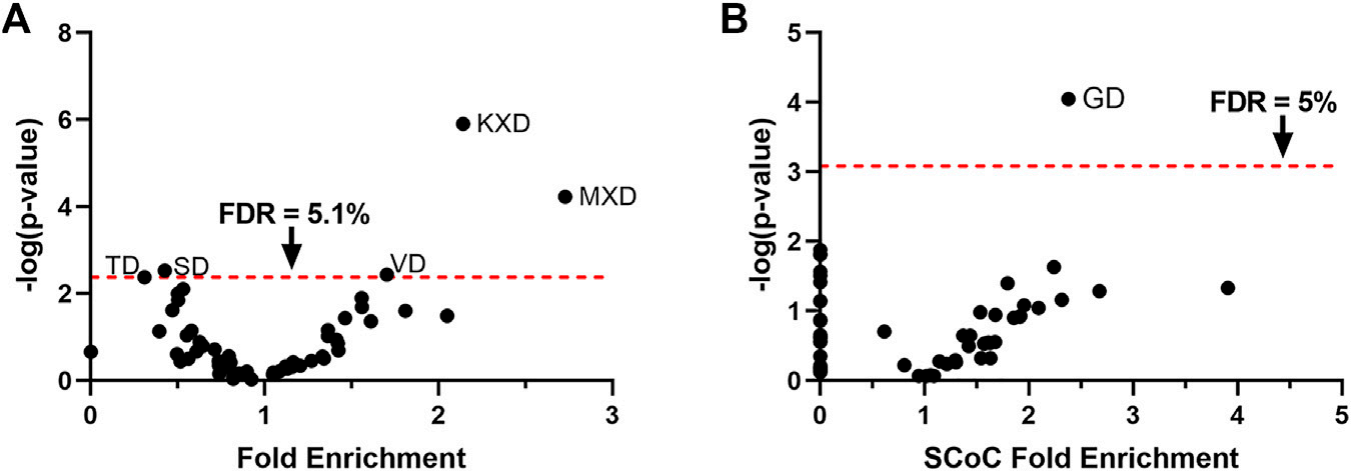
The GD motif is enriched among SCoC substrates in the apoptotic chick auditory brainstem, despite preference for the apoptotic VD motif. **(A)** Hypergeometric tests were used to test proteolytic preference in the apoptotic chick auditory brainstem among the 60 P4-P2 motifs. Consistent with apoptotic activity, we detected enrichment for the VD motif. The KXD and MXD motifs were also enriched. We found no significant enrichment for the motifs that made up the caspase consensus sequence of the non-apoptotic auditory brainstem. **(B)** Monte Carlo analyses were used to identify motifs associated with SCoC substrates. Only the GD motif was significantly enriched among SCoC protein-cleavages, identifying it as the most ideal candidate for caspase activity directed toward SCoC proteins. FDR: False Discovery Rate, derived by Benjamini, Krieger, and Yekutieli correction.

We next sought to test whether the enrichment for specific motifs among cytoskeletal substrates is unique to non-apoptotic caspase activity, or whether such enrichment might be observed in apoptotic sites as well. The chick auditory brainstem undergoes apoptotic cell death starting on embryonic day (E) 12 or 13 (Rotschafer et al., 2016), with a loss of many cells in auditory nuclei including NM and NL (Rubel et al., 1976). These are the same cells in which we observed non-apoptotic caspase activity just a few days earlier on E10 and from which our set of non-apoptotic caspase substrates was derived. We therefore conducted tandem mass spectrometry on E13 brainstems as an apoptotic counterpart to the E10 non-apoptotic auditory brainstem. As with the non-apoptotic brainstem proteome, we filtered for peptides C-terminal to a D↓X terminus, which yielded 199 distinct caspase cleavage sites from 126 distinct proteins (Supplementary Data S3). We used hypergeometric tests to reveal proteolytic preference for the 60 P4-P2 motifs (Figure 3A) and found significant enrichment for three motifs: KXD (Fold enrichment: 2.14; *p* = 1.3 × 10^–6^; *q* = 7.6 × 10^–5^), MXD (Fold enrichment: 2.73; *p* = 5.9 × 10^–5^; *q* = 1.8 × 10^–3^), and VD (Fold enrichment: 1.70; *p* = 3.7 × 10^–3^; *q* = 0.051), corresponding to a consensus sequence of (K/M)VD. After correction for multiple comparisons, we detected no significant enrichment in the motifs that comprised the chick non-apoptotic consensus sequence: IXXD (Fold enrichment: 1.13; *p* = 0.53; *q* = 0.66), GD (Fold enrichment: 1.46; *p* = 0.036; *q* = 0.16), and RD (Fold enrichment: 0.76; *p* = 0.39; *q* = 0.59). Since we were primarily concerned with the role of the P2 subsite in determining cleavage site preference, we interpreted the heightened preference for the apoptotic motif VD in the E13 auditory brainstem as evidence for activity resembling the canonical apoptotic consensus sequence at the P2 subsite.

We next aimed to identify motifs associated with SCoC protein-cleavages in the chick apoptotic degradome. We subjected all 60 P2-P4 motifs to Monte Carlo analyses in which cleavage events were simulated at their observed frequencies for each motif in each replicate. Fold enrichment of SCoC protein-cleavages and *p*-values were obtained for each motif by comparing the observed SCoC protein-cleavages at the motif to the expected distribution of SCoC protein-cleavages at the motif (Figure 3B). We found that SCoC protein-cleavages were significantly enriched for a single motif: GD (Fold Enrichment: 2.38; *p* = 8.9 × 10^–5^, *q* = 5.4 × 10^–3^). GD was part of the consensus sequence in the E10 non-apoptotic auditory brainstem, suggesting that the non-apoptotic consensus sequence represents a departure from the apoptotic consensus sequence toward a motif associated with SCoC proteins (*p* = 0.025; hypergeometric test for the SCoC-enriched motif being from the non-apoptotic consensus sequence). All three SCoC proteins (actin, alpha tubulin, and beta tubulin) observed in the three replicate samples of the E13 brainstem were found to be cleaved at a GD site, so a double-motif analysis was not warranted for this dataset. Additionally, all 17 distinct SCoC cleavage sites in the E13 apoptotic brainstem were also observed in the E10 non-apoptotic brainstem, but 23 distinct SCoC cleavage sites were only found in the E10 brainstem, indicating that non-apoptotic caspase activity cleaves a mix of apoptotic and novel SCoC cleavage sites. These findings corroborate the function of the GD motif in facilitating specific caspase-dependent proteolysis of SCoC proteins in non-apoptotic contexts.

### Prediction of the Neurodevelopmental Non-apoptotic Consensus Sequence in Humans

**FIGURE 4:**
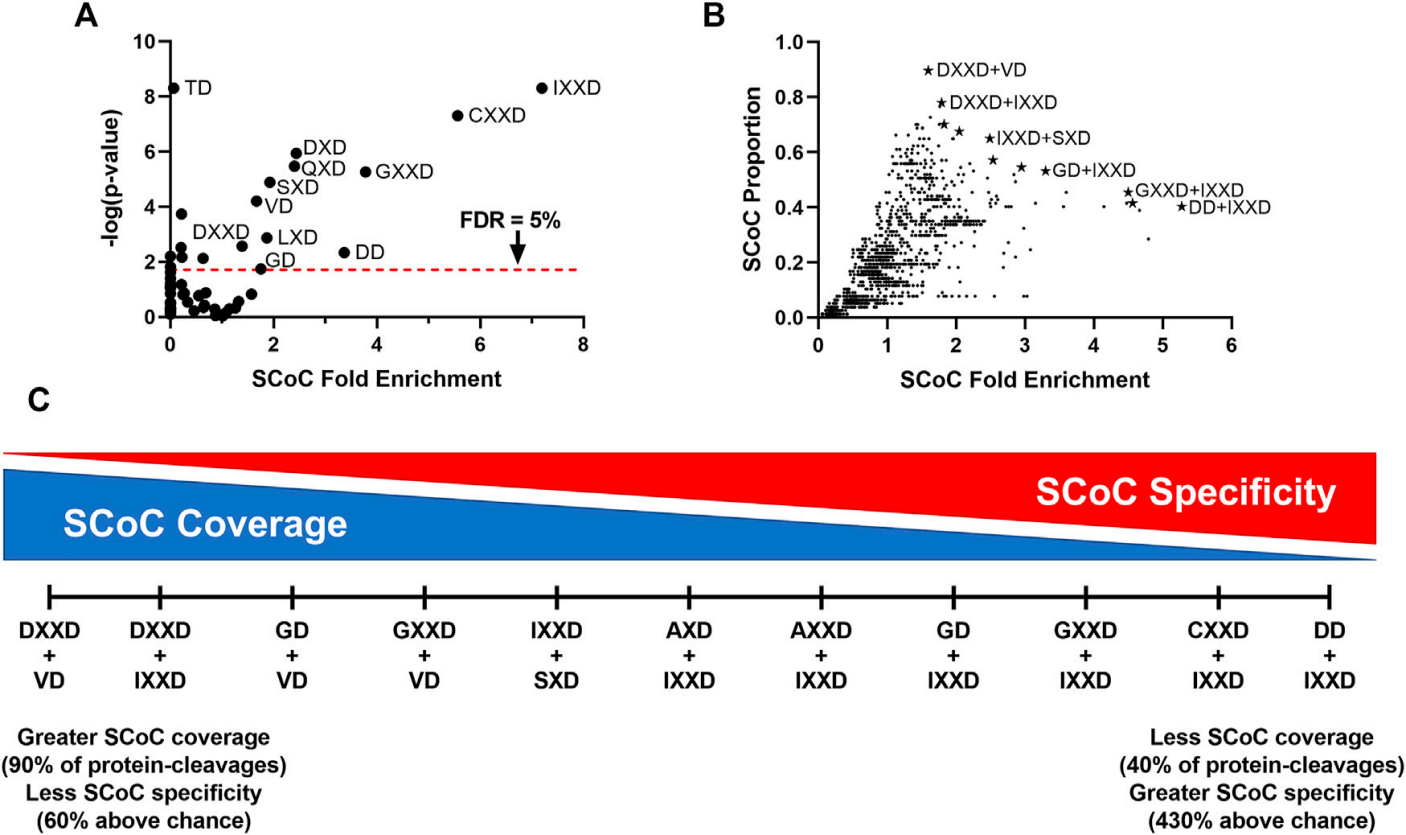
SCoC-specific motifs in the Degrabase represent likely candidates for human non-apoptotic consensus sequences. **(A)** To identify motifs associated with SCoC protein-cleavages, Monte Carlo analyses were used to simulate cleavage events at each P4-P2 motif in SCoC caspase substrates from the Degrabase, a database of cleavage events in human cell lines. The observed protein-cleavage count for each motif was compared to the random distribution of expected protein-cleavages to calculate SCoC fold enrichment and *p*-values. We identified 11 motifs that were significantly associated with SCoC protein-cleavages, including 2 motifs present in the chick non-apoptotic consensus sequence (GD and IXXD). *p*-values for IXXD and TD are maximum estimates, as none of the 10 million Monte Carlo simulations yielded the observed protein-cleavages for these motifs. **(B)** As with single motifs, Monte Carlo analyses were used to estimate SCoC fold enrichment and *p*-values for observed protein-cleavages of non-redundant motif combinations. The proportion of observed SCoC protein-cleavages for each motif was plotted against the SCoC fold enrichment to identify motif combinations likely to be preferred in human non-apoptotic caspase-dependent processes (starred datapoints). These 11 candidates were selected in a stepwise fashion, beginning with the motif combination that had the highest SCoC fold enrichment and including additional motifs with lower SCoC fold enrichment only if they also had a higher SCoC protein-cleavage proportion. Labels of select candidates are shown. All 11 candidates had *p*-values below the 5% family-wise error rate cutoff, 2.8 × 10^–5^. Motif combinations of IXXD, CXXD, or GXXD and any motif with 0 or 1 observed protein-cleavages are not shown, as these combinations tended to have artificially inflated fold enrichment values despite being unlikely candidates for an SCoC-directed consensus sequence. **(C)** The 11 motif combinations with maximum SCoC protein-cleavage proportions for their given SCoC fold enrichment represent a spectrum with a tradeoff between high coverage of SCoC protein-cleavages and high specificity for SCoC protein-cleavages. FDR, False Discovery Rate, derived by Benjamini, Krieger, and Yekutieli correction.

Caspases exhibit remarkable conservation between species, so we next explored the possibility that human caspases are also capable of shifting to an SCoC-specific consensus sequence in non-apoptotic contexts. No proteome for a non-apoptotic caspase-dependent process in humans has yet been published, but the Degrabase (Crawford et al., 2013) offers a large sample of apoptotic cleavage sites that might be interrogated via the same method we used to show that the GD motif is associated with SCoC protein-cleavages both in the E10 and E13 chick auditory brainstem. Degrabase contains 1,684 distinct D↓X sites, observed a total of 5,713 times in 32 biological replicates and belonging to 1,267 distinct Uniprot accessions (Supplementary Data S4). To identify which motifs are disproportionately associated with SCoC protein-cleavages, we conducted Monte Carlo analyses on the 60 P2-P4 motifs. As before, we built an expected distribution of the number of SCoC protein-cleavages for each motif by modeling proteolysis of each SCoC protein at the observed frequency of the motif in that replicate. We summed the results of all replicates and compared this distribution to the observed protein-cleavages for each motif, obtaining fold enrichment and *p*-values as before (Figure 4A). We found that 11 motifs were significantly associated with SCoC protein-cleavages in the Degrabase at a 5% false discovery rate. Notably, two motifs found in the chick non-apoptotic consensus sequence (IXXD and GD) were among these 11 (*p* = 0.044; hypergeometric test for 2 or more of the 11 motifs being from the chick non-apoptotic consensus sequence).

Despite several motifs conveying substantial preference for SCoC proteins (more than 7-fold in the case of IXXD), no single motif accounted for the majority of SCoC protein-cleavages in Degrabase. We therefore sought to identify double-motif combinations that provide better coverage of SCoC protein-cleavages while maintaining specificity of proteolysis. As with the single motifs, we ran Monte Carlo simulations on the 1770 double-motif combinations. To find likely candidates for an SCoC-directed non-apoptotic consensus sequence, we plotted the proportion of SCoC protein-cleavages attributable to each motif combination against the fold enrichment of SCoC protein-cleavages at each motif combination (Figure 4B). We first selected the motif combination with the highest SCoC fold enrichment (DD + IXXD) as a non-apoptotic consensus candidate. All other candidates were permitted to have a lower SCoC fold enrichment only if they also had a higher SCoC proportion than the previous candidate. This stepwise method yielded 11 motif combinations that represented a spectrum of consensus sequence candidates ranging from high specificity of SCoC proteolysis to high coverage of SCoC protein-cleavages (Figure 4C). All 11 of these candidates had *p*-values consistent with a family-wise error rate of less than 5% (*p* < 2.8 × 10^–5^). Remarkably, one of the candidates, GD + IXXD, resembled the consensus sequence of the non-apoptotic chick auditory brainstem, indicating that the cleavage site preference in non-apoptotic caspase-dependent processes may be conserved among vertebrates (*p* = 9.3 × 10^–3^; hypergeometric test for 1 or more of the 11 candidates containing 2 motifs from the chick non-apoptotic consensus sequence).

## Discussion

How caspases select their substrates is key to understanding how non-apoptotic caspase-dependent processes can shape cell fate non-lethally. Here we showed that non-apoptotic caspase activity prior to programmed cell death in the chick auditory brainstem targets a non-canonical consensus sequence, IX(G/R)D↓. Portions of this consensus sequence (especially the GD motif) were associated with the GO term “Structural Constituent of Cytoskeleton” in the E10 non-apoptotic chick brainstem, as well as the E13 apoptotic chick brainstem and the human apoptotic degradome, suggesting a conserved function of the cleavage site sequence in guiding caspase activity toward cytoskeletal substrates.

### Cytoskeleton-Directed Caspase Activity Provides a Mechanism by Which Caspases can Alter Cell Morphology

Our findings thus elucidate a mechanism by which caspases can specifically cleave cytoskeletal subunits, a function needed for many of their non-lethal neurodevelopmental roles. Studies on non-apoptotic neurodevelopmental roles of caspases in *Drosophila* have provided abundant evidence that axonal growth cones use local changes in caspase activity as a mechanism linking external guidance cues to internal cytoskeletal changes (reviewed in Kellermeyer et al., 2018). A variety of non-apoptotic functions for caspases in vertebrate neural circuit formation have been enumerated as well, including growth cone chemotaxis (Campbell and Holt, 2003), axon outgrowth (Westphal et al., 2010), axon branching (Campbell and Okamoto, 2013), dendritic pruning (Ertürk et al., 2014), dendritic branching (Khatri et al., 2018), and long-term depression (Li et al., 2010; Han et al., 2013). However, few studies (Westphal et al., 2010; Sokolowski et al., 2014; Khatri et al., 2018) have demonstrated that caspases directly cleave cytoskeletal proteins to carry out their neurodevelopmental roles, let alone explained how caspases avoid cleaving pro-apoptotic substrates in the process. Our study is the first to provide evidence not only that cytoskeletal proteins are cleaved in non-apoptotic caspase-dependent processes, but also that the cleavage site preference of caspases is shifted toward sites that favor degradation of cytoskeletal proteins during neural circuit formation.

### Limitations

Our method for determining caspase consensus sequences assumes that all aspartates within caspase substrates are equally susceptible to caspase-mediated proteolysis. However, since caspases most readily cleave residues with cytosolic topology and with high solvent accessibility (Soni and Hardy, 2021), this assumption could be improved in future studies by only considering aspartates that meet these criteria. Transmembrane proteins would be most affected by this change, since caspases do not usually operate in the luminal or extracellular space, albeit some exceptions have been observed (Hentze et al., 2001; Shamaa et al., 2015; Wang et al., 2016). Despite this drawback, the frequency of P2-P4 motifs in the entire set of substrates is likely to be a good approximation for the frequency of motifs accessible to caspase activity.

Our analysis is also constrained by the progression of caspase activity in the developing auditory brainstem. Non-apoptotic caspase activity assists in auditory brainstem circuit formation from E6 to E12, while apoptotic cell death predominates from E12 to E17 (Rubel et al., 1976; Rotschafer et al., 2016). However, it is unknown whether non-apoptotic caspase activity continues to refine auditory brainstem synapses during the period of cell death. If it does, our E13 caspase degradome may be derived from simultaneous caspase-dependent processes instead of apoptosis alone. Nevertheless, our analysis benefits from the use of a system in which apoptosis is not artificially induced but rather reflects normal developmental cell death. The different caspase consensus sequences in the E10 and E13 auditory brainstems also suggest that a distinct set of substrates is being targeted at the two ages, especially because the apoptotic VD motif was part of the E13 consensus sequence.

### Preference for IXXD During Non-apoptotic Processes May Prevent Proteins From Being Cleaved at Pro-apoptotic Sites

Although we identified IXXD in the human caspase degradome by searching for motifs more likely to be cleaved in SCoC proteins, IXXD was not enriched among SCoC protein-cleavages in the non-apoptotic chick auditory brainstem. Indeed, we found no GO terms enriched among substrates cleaved at IXXD compared to all auditory brainstem substrates. This discrepancy may reflect inter-species differences in SCoC-associated motifs. However, if IXXD is not involved in directing caspase activity toward any functional category of substrates in the non-apoptotic chick auditory brainstem, what is the significance of this motif? One possibility is that preference for IXXD protects cells from potentially lethal cleavage events. Negatively-charged P4 residues (i.e., DXXD or EXXD motifs) are very common in the cleavage sites of major pro-apoptotic substrates (Thornberry et al., 1997; Fischer et al., 2003; Crawford et al., 2013; Rawlings et al., 2018). Even SCoC proteins, which are not canonical apoptotic signaling molecules, can promote apoptosis when cleaved at sites with negatively charged P4 residues. Caspase-mediated proteolysis of actin at ELPD^244^ or vimentin at DSVD^85^ yields pro-apoptotic cleavage fragments, the overexpression of which is sufficient to cause morphological changes consistent with cell death (Mashima et al., 1999; Byun et al., 2001). The preference for hydrophobic isoleucine at the P4 position in the IXXD motif may thus serve to prevent proteolysis at sites likely to lead to the cell’s demise. This anti-apoptotic cleavage site preference joins the ranks of other such mechanisms that prevent cell death during non-apoptotic caspase-dependent processes, including binding by Inhibitors of Apoptotic Proteins (IAPs), which act as muzzles for the caspase active site (Scott et al., 2005; Silke and Meier, 2013); localization to cellular compartments far from the cell body and nucleus, such as growth cones and dendrites (Campbell and Holt, 2003; Li et al., 2010); low expression levels of pro-apoptotic molecules besides caspases (Wright et al., 2004; Hollville and Deshmukh, 2017; Mukherjee and Williams, 2017); and rapid degradation of active caspases, a coordinated effort of IAPs and the proteasome (Galbán and Duckett, 2010; Ertürk et al., 2014).

### The Non-apoptotic Auditory Brainstem Likely has a Greater Range of Caspase Activity Than the Apoptotic Auditory Brainstem

Our data show that the E10 auditory brainstem has more distinct caspase cleavage sites and substrates (655 and 365, respectively) compared to the E13 auditory brainstem (191 and 126, respectively), suggesting that there is more caspase activity in the non-apoptotic auditory brainstem than during apoptosis. However, this observation does not imply a greater density of caspase activity (per cell or per unit volume) on E10 than on E13. Many NM axons contain cleaved caspase-3 on E10 (Rotschafer et al., 2016), while relatively few NM and NL cells undergo apoptosis on E13 (Rubel et al., 1976). The greater number of non-apoptotic cleavage sites might therefore reflect the greater number of cells with active caspases, protected by the usual mechanisms of preventing non-apoptotic caspase activity from turning lethal, described above. It is also possible that developing neurites contain a greater variety of potential caspase substrates than do cell bodies and nuclei, resulting in greater diversity in caspase cleavage sites and substrates during non-apoptotic caspase activity than during apoptosis.

### Caspase-3 Probably Produces Most of the Observed Cleavage Sites in Both the Non-apoptotic and Apoptotic Auditory Brainstem

Though the caspase family exhibits a range of cleavage site preferences, (Thornberry et al., 1997; Stennicke et al., 2000; Julien et al., 2016; Rawlings et al., 2018), it is unlikely that the different consensus sequences we observed in apoptotic and non-apoptotic contexts are attributable to the activity of distinct caspases. Indeed, there is evidence that caspase-3 activity predominates in the Degrabase and in the chick brainstem at E10 and E13. The creators of Degrabase note that the apoptotic consensus sequence, DEVD↓, is identical to the individual preferences of caspase-3, and caspase-7 in positional scanning peptide libraries, consistent with these caspases’ roles as apoptotic executioners (Crawford et al., 2013). Caspase-3 has been shown to have a broader substrate repertoire than caspase-7 *in vitro* (Walsh et al., 2008), and caspase-3 is the only apoptotic executioner required for major apoptotic outcomes (Slee et al., 2001), suggesting that Degrabase predominately reflects caspase-3 activity. Besides caspase-3, we detected three additional caspases (-6, -8, and -9) in the non-apoptotic chick auditory brainstem (Weghorst et al., 2020) and two additional caspases (-1 and -2) in the apoptotic auditory brainstem. None of these caspases exhibits substantial preference for the GD or RD motifs in synthetic peptides, so the P2 position in the non-apoptotic consensus sequence cannot be easily explained with reference to a specific caspase’s known activity. Additionally, only caspase-3 was detected in every single biological replicate, and at much higher Mascot scores than every other caspase, suggesting that caspase-3 is the most abundant caspase in both the apoptotic and non-apoptotic chick auditory brainstem (Weghorst et al., 2020). Caspases, like all enzymes, are catalytic, so we cannot rule out the possibility that another less-abundant caspase contributes some of the observed caspase cleavage sites. However, caspase-3 is the most active caspase even at equal abundances (Julien et al., 2016), so it is likely responsible for most of the observed caspase cleavage sites in the auditory brainstem, as in Degrabase.

### Post-Translational Modifications to Caspase-3 Are Likely to Underlie the Non-canonical Caspase Consensus Sequence of the Non-apoptotic Auditory Brainstem

If caspase-3 is the primary aspartate-directed protease in all contexts examined here, how is it capable of such distinct cleavage site sequence preferences? One possibility is that the optimal cleavage site sequence is altered by allosteric or post-translational modifications (PTMs) to caspase-3 itself (Agniswamy et al., 2007; Hardy and Wells, 2009; Häcker et al., 2011; Cade et al., 2014; Dagbay et al., 2014; Maciag et al., 2016; Eron et al., 2017; Zamaraev et al., 2017; Herr, 2018). Relatively few amino acid substitutions are required to change the P4 subsite preference of caspase-3 and -7 from the negatively-charged aspartate (i.e. DXXD) to a preference for hydrophobic residues such as isoleucine and valine (Hill et al., 2016; Bingöl et al., 2019; Grinshpon et al., 2019; Yao et al., 2021). Similar PTM-mediated changes to the caspase-3 active site may play a role in shifting the P4 consensus motif to IXXD, which we observed in the non-apoptotic chick auditory brainstem and which we predict is the most likely non-apoptotic consensus motif in humans. Modulatory PTMs of caspase substrates are common as well, but these are unlikely to produce systematic changes in cleavage site preference (Tözsér et al., 2003; Dix et al., 2012; Turowec et al., 2014; Kumar and Cieplak, 2018; Thomas et al., 2018; Maluch et al., 2021). The specific PTMs responsible for the novel caspase consensus sequence observed in the non-apoptotic chick auditory brainstem remain to be determined.

## Data Availability

The datasets presented in this study can be found in online repositories. The mass spectrometry proteomics data have been deposited to the ProteomeXchange Consortium via the PRIDE partner repository, with the dataset identifier PXD030697.
